# Prognostic value of long non-coding RNA signatures in bladder cancer

**DOI:** 10.18632/aging.102185

**Published:** 2019-08-20

**Authors:** Anbang He, Shiming He, Ding Peng, Yonghao Zhan, Yifan Li, Zhicong Chen, Yanqing Gong, Xuesong Li, Liqun Zhou

**Affiliations:** 1 Department of Urology, Peking University First Hospital, Beijing 100034, China; 2 Institute of Urology, Peking University, Beijing 100034, China; 3 National Urological Cancer Center, Beijing 100034, China; 4 Beijing Key Laboratory of Urogenital Diseases (Male) Molecular Diagnosis and Treatment Center, Beijing 100034, China

**Keywords:** bladder cancer, lncRNA, lasso, OS, RFS

## Abstract

Bladder cancer (BLCA) is a devastating cancer whose early diagnosis can ensure better prognosis. Aim of this study was to evaluate the potential utility of lncRNAs in constructing lncRNA-based classifiers of BLCA prognosis and recurrence. Based on the data concerning BLCA retrieved from TCGA, lncRNA-based classifiers for OS and RFS were built using the least absolute shrinkage and selection operation (LASSO) Cox regression model in the training cohorts. More specifically, a 14-lncRNA-based classifier for OS and a 12-lncRNA-based classifier for RFS were constructed using the LASSO Cox regression. According to the prediction value, patients were divided into high/low-risk groups based on the cut-off of the median risk-score. The log-rank test showed significant differences in OS and RFS between low- and high-risk groups in the training, validation and whole cohorts. In the time-dependent ROC curve analysis, the AUCs for OS in the first, third, and fifth year were 0.734, 0.78, and 0.78 respectively, whereas the prediction capability of the 14-lncRNA classifier was superior to a previously published lncRNA classifier. As for the RFS, the AUCs in the first, third, and fifth year were 0.755, 0.715, and 0.740 respectively. In summary, the two-lncRNA-based classifiers could serve as novel and independent prognostic factors for OS and RFS individually.

## INTRODUCTION

Bladder cancer (BLCA) is the ninth most common malignant cancer with high incidence and recurrence rates [1, 2]. The risk evaluation of prognosis and recurrence has a critical impact on clinical decision and patient consultation [3]. The most significant factors involved in this evaluation include general condition of patients, clinicopathological characteristics, clinical treatment and progression of disease [1, 4, 5]. Additionally, tumor node metastasis (TNM) staging system, is currently applied in clinical work as the most common prediction tool [4, 6]. Nevertheless, this single clinical prediction model is considered less accurate at prediction than models merging several clinical characteristics [7]. Moreover, the current clinical prediction model cannot facilely incorporate novel factors, such as molecular biomarkers and complex external environmental factors [5].

Over the years, scientists have proposed numerous potential molecular signatures as predictors of the risk of cancer progression, with the most important of them being the DNA methylation-based models [8–10], mRNA [11, 12], microRNA(miRNA) [13] and long non-coding RNA (lncRNA)-based models [14, 15]. Increasing evidence has indicated the critical role of lncRNAs in BLCA prognosis and recurrence, being involved in cancer initiation, progression and metastasis [16]. However, the prognostic value of lncRNAs in BLCA has not been adequately explored yet.

In this study, in an effort to assess the potential utility of lncRNAs in prognosis and recurrence of BLCA, we constructed a 14-lncRNA-based classifier for overall survival (OS) and a 12-lncRNA-based classifier for relapse-free survival (RFS) by using the least absolute shrinkage and selection operation (LASSO) Cox regression. Both of the lncRNA-based classifiers could optimize the predictivity of the current TNM staging system. Our results demonstrate that these lncRNA-based classifiers could be used as reliable prognostic predictors of BLCA survival and recurrence.

## RESULTS

### Data source and processing

**Figure 1:**
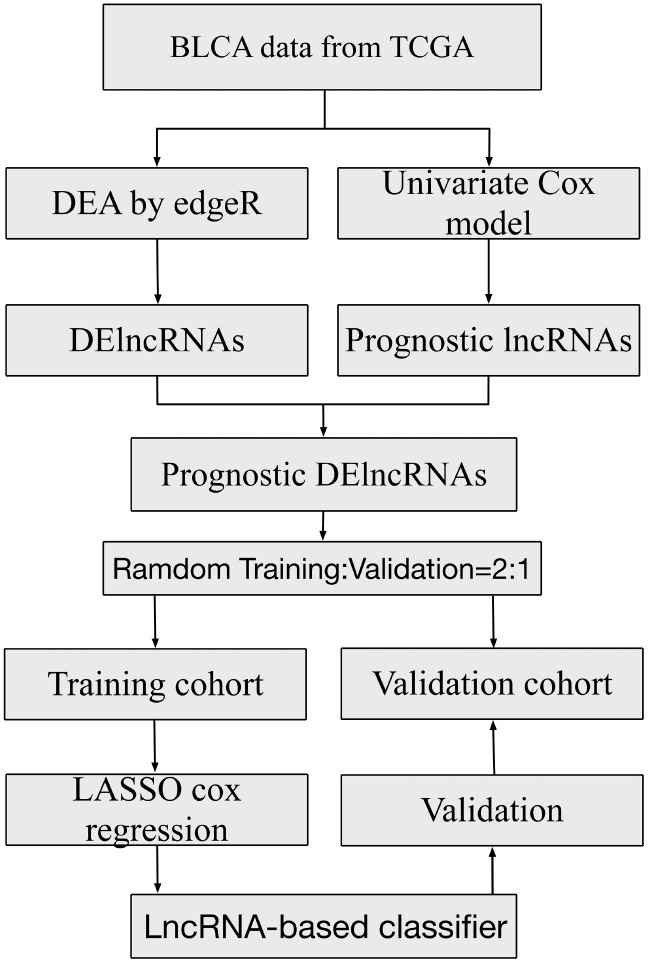
**Study flowchart showing steps involved in construction of lncRNA-based prognostic signatures.**

**Figure 2:**
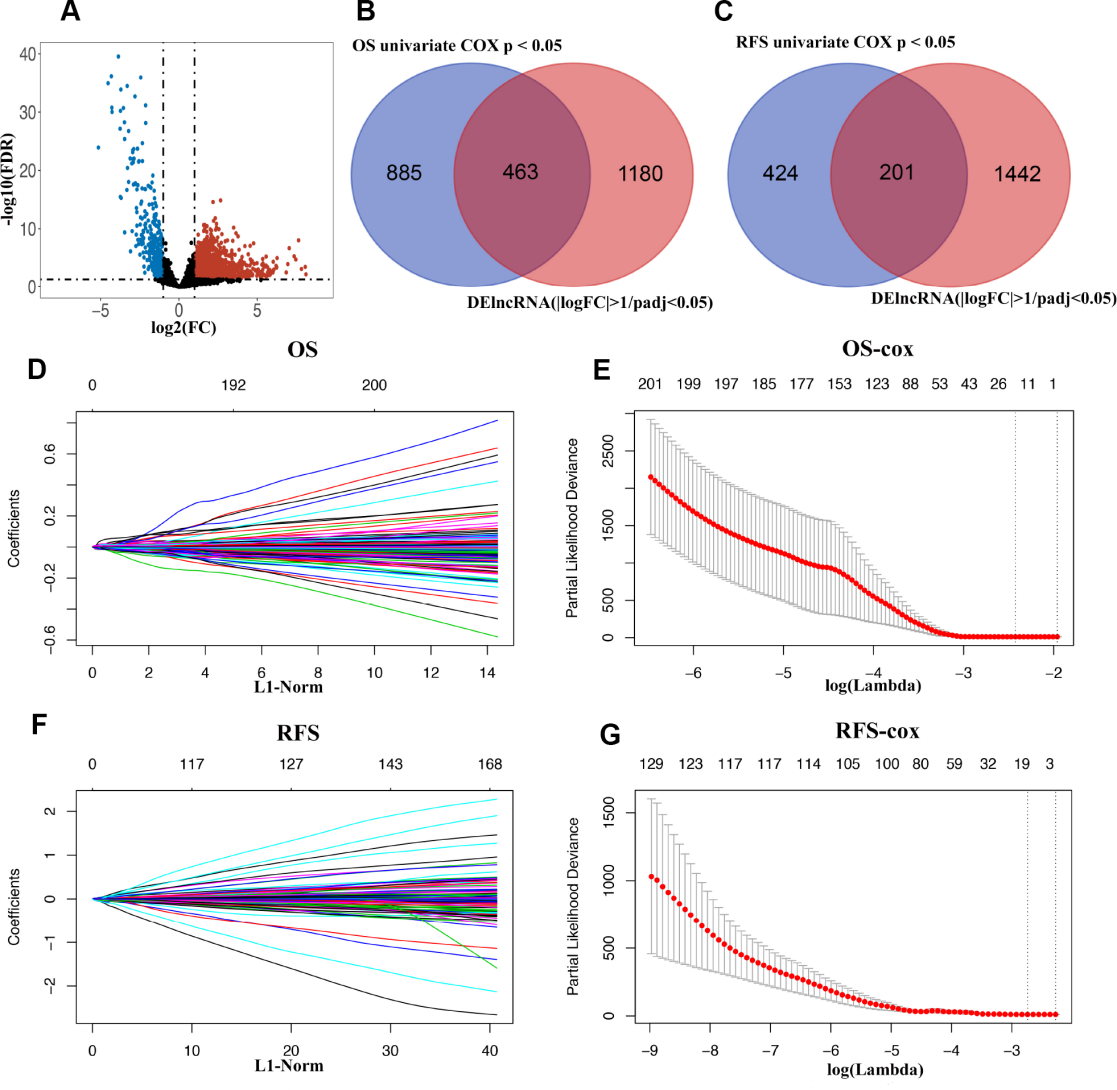
(**A**) Volcano plot of differentially expressed lncRNAs in TCGA-BLCA cohort. (**B** and **C**) Venn diagram of prognostic DElncRNAs in prognostic lncRNAs (OS/RFS univariate cox p < 0.05) and DElncRNAs(|logFC| >1 and padj < 0.05). (**D**) 20-time cross-validation for tuning parameter selection in the LASSO model for OS. (**E**) LASSO coefficient profiles of 463 prognostic DElncRNAs for OS. (**F**) 20-time cross-validation for tuning parameter selection in the LASSO model for RFS. (**G**) LASSO coefficient profiles of 201 prognostic DElncRNAs for RFS.

The lncRNA expression profiles in BLCA tissues (n=414) along with the adjacent non-tumor tissues (n=19) were obtained from the TCGA database. As shown in Figure 1, a total of 1643 DElncRNAs (Figure 2A) with |logFC| >1 and padj < 0.05 were identified using edgeR. Additionally, lncRNAs with *p* < 0.05 were chosen by applying a univariate Cox regression in the entire data. Following this, 463 lncRNAs (OS, Figure 2B) and 201 lncRNAs (RFS, Figure 2C) were retained for the next step of the analysis. For OS, these samples (n=406) were randomly split into training (n=271) and validation sets (n=135) at 2:1 ratio. Similarly, for RFS, the samples (n=337) were randomly split into training (n=225) and validation sets (n=112) at a 2:1 ratio. The LASSO Cox selection method was applied to construct the prognosis-predicting models in the training cohort at a 20-fold cross-validation (OS: Figure 2D, 2E; RFS: Figure 2F, 2G).

### Construction of lncRNAs classifiers for OS and RFS

**Figure 3:**
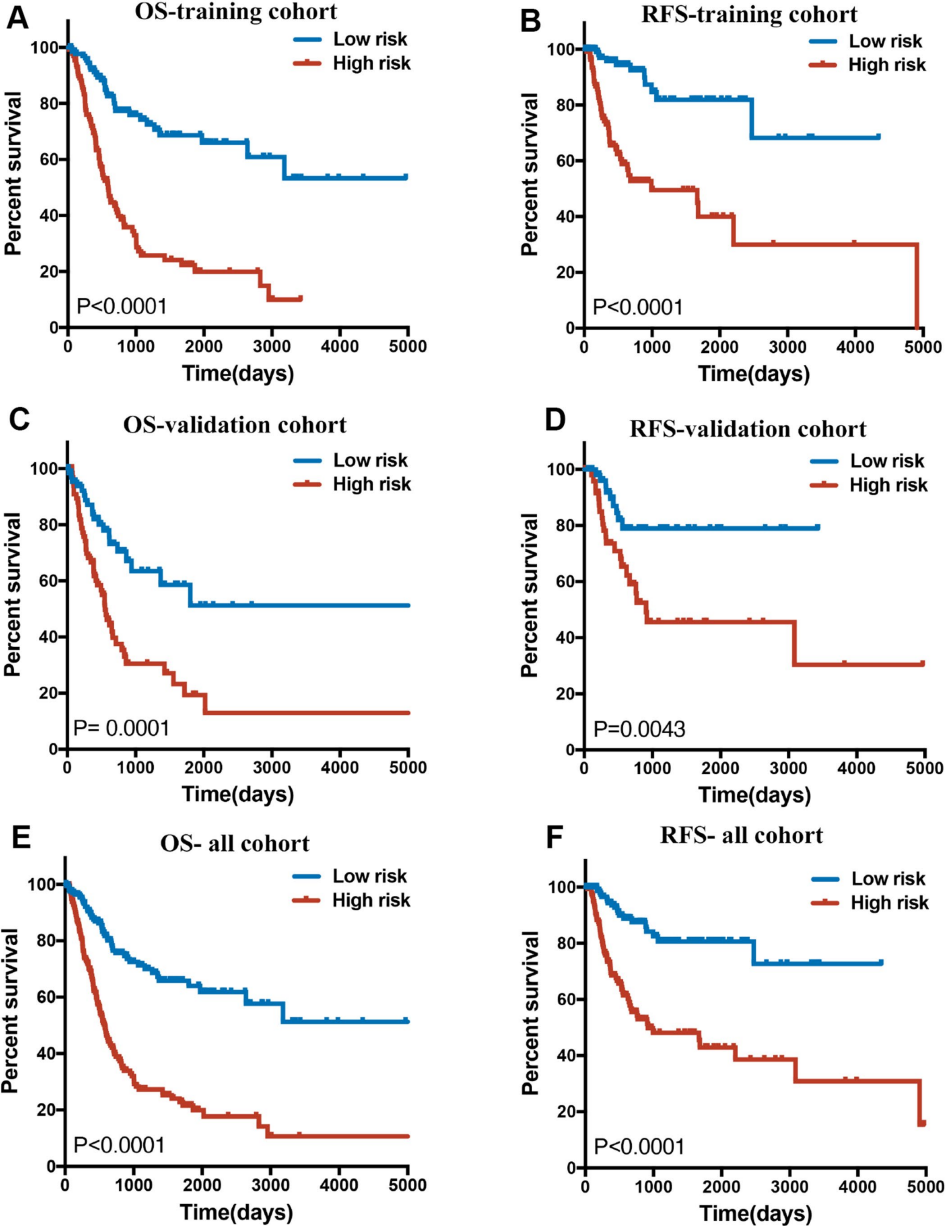
(**A**, **C** and **E**) Overall survival curves of BLCA patients in training, validation and all cohorts with a low or high risk of death, according to 14-lncRNA-based classifier risk score level. (**B**, **D** and **F**): Relapse-free survival curves of BLCA patients in training, validation and all cohorts with a low or high risk of death, according to 12-lncRNA-based classifier risk score level.

In the training cohort, a 14-lncRNA-based classifier for OS and a 12-lncRNA-based classifier for RFS were constructed using the LASSO Cox regression mode at 20-fold cross-validation. Detailed information of these lncRNAs is shown in Table 1. According to the prediction value, patients were divided into high- and low-risk groups based on the cut-off of the median risk score. The Kaplan–Meier log-rank test showed significant differences in OS and RFS between low- and high-risk groups in the training cohorts (Figure 3A, 3B), the validation cohorts (Figure 3C, 3D) and in the whole cohorts (Figure 3E, 3F).

**Table 1:** The detailed information of lncRNAs for constructing the prognostic signature.

**14-lncRNA-based classifier for OS**					
**Gene name**	**ENSG_ID**	**Chromosome**	**Gene start (bp)**	**Gene end (bp)**	**β**
AL662844.4	ENSG00000272501.1	6p21.33	31195200	31198037	0.000859567
MAFG-AS1	ENSG00000265688	17q25.3	81927829	81930753	0.00024963
RNF144A-AS1	ENSG00000228203	2p25.1	6918682	6912276	0.00135716
AC093788.1	ENSG00000273449	4q32.2	163529771	163530697	0.001168141
AC024060.1	ENSG00000271870	3p26.2	3152942	3153435	0.000445531
LINC01138	ENSG00000274020	1q21.2	148459920	148432959	0.000350856
Z84484.1	ENSG00000224666	6p21.31	36386831	36393462	0.002095112
MANCR	ENSG00000231298	10p15.1	4650185	4678154	0.000322206
AL590428.1	ENSG00000231652	6q13	73693903	73696131	0.004351042
CERS3-AS1	ENSG00000259430	15q26.3	100372939	100437914	0.003812687
AL590999.1	ENSG00000235033	6p21.2	39881804	39900071	0.000167192
Z98200.1	ENSG00000271734	6q21	108030249	108030718	0.003081411
LINC01169	ENSG00000259471	15q22.31	66582190	66685798	0.002831088
AL049775.1	ENSG00000205562	14q31.3	85530313	85522055	0.002947469
**12-lncRNA-based classifier for RFS**					
**Gene name**	**ENSG_ID**	**Chromosome**	**Gene start (bp)**	**Gene end (bp)**	**β**
NALCN-AS1	ENSG00000233009	13q32.3	100708325	101059286	0.003081179
AL353593.2	ENSG00000269934	1q42.13	228274584	228276066	0.007001554
AC116914.2	ENSG00000262692	17p13.2	3721628	3722488	0.000160626
AC092910.3	ENSG00000242622	3q13.33	120094895	120136783	0.00432904
FLJ22447	ENSG00000232774	14q23.1	61570540	61658696	0.000201789
SH3RF3-AS1	ENSG00000259863	2q13	109127327	109128930	0.006699057
AL121658.1	ENSG00000272716	2p22.3	32165046	32165757	0.005552396
AL590428.1	ENSG00000231652	6q13	73693903	73696131	0.003681168
AC080013.3	ENSG00000271778	3q25.32	158782547	158783124	0.001601851
LSAMP-AS1	ENSG00000240922	3q13.31	116360024	116370090	0.011192555
SLC26A4-AS1	ENSG00000233705	7q22.3	107653968	107662151	0.002233053
AC023051.1	ENSG00000234428	12p11.23	26623369	26649479	0.011428433

### Correlation between lncRNAs classifiers and clinicopathologic characteristics

**Figure 4:**
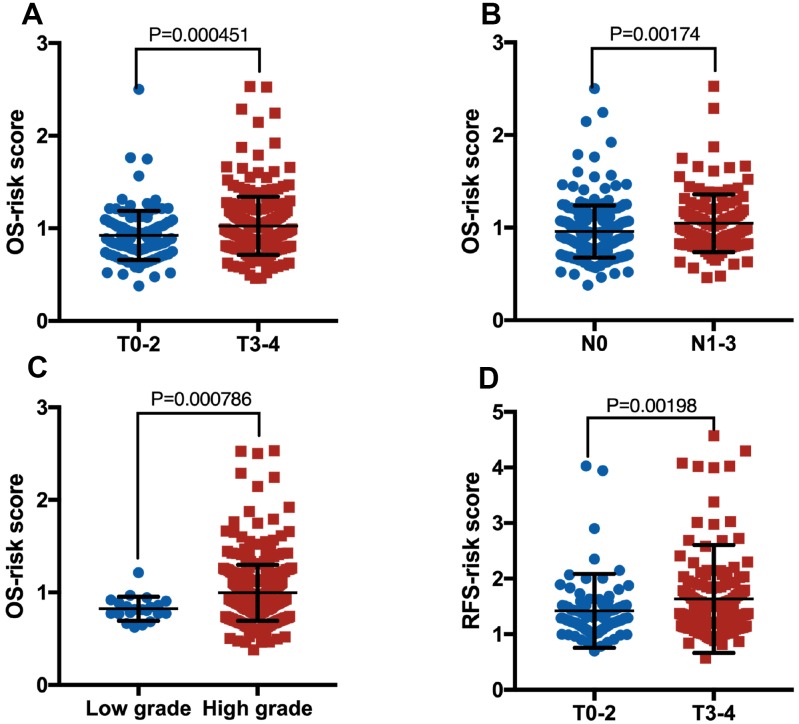
Boxplot of risk score in patients with pT (**A**, OS), pN (**B**, OS), grade (**C**, OS) and pT (**D**, RFS).

There were no significant difference and deviation between the training cohort and the validation cohort, because these samples were randomly split into training and validation sets at a 2:1 ratio in Tables 2–5. As shown in Table 2, for OS, the clinical characteristics (subtype, pT, pN and grade) showed significant differences between the two groups in whole cohort. However, for RFS, many clinical characteristics, except pT, did not vary significantly between the two groups in whole cohort (Table 3). Though the lncRNA-based risk scores of OS or RFS were independent of several clinical characteristics, positive associations were detected between them (Figure 4). Patients with high pT, pN or grade were inclined to have a high-risk score.

**Table 2:** Correlations between risk score of the 14-marker-based classifier with OS and clinicopathological characteristics in training cohort, validation cohort and whole cohort.

**Parameters**	**High risk**	**Low risk**	**Pearson *x*2**	**P**
Training cohort				
Age			0.06006	0.8064
>60	102	101		
≤60	33	35		
Gender			1.336519	0.247649
male	97	106		
female	38	30		
–Subtype			6.471522	**0.010962**
Papillary	37	58		
Non-Papillary	96	78		
pT			4.199471	**0.040437**
T3-4	93	75		
T0-2	35	49		
pN			0.411615	0.521151
N1-3	39	35		
N0	82	88		
pM			1.633899	0.502242
M1	0	2		
M0	62	75		
Grade			6.48751	**0.010864**
high	131	3		
low	123	13		
Validation cohort				
Age			0.141667	0.70663
>60	49	47		
≤60	19	21		
Gender			1.314715	0.251543
male	46	52		
female	22	16		
Subtype			8.421529	**0.003708**
Papillary	10	25		
Non-Papillary	56	42		
pT			3.986205	**0.045874**
T3-4	48	35		
T0-2	15	24		
pN			9.125692	**0.00252**
N1-3	36	19		
N0	25	41		
pM			2.92108	0.087429
M1	6	3		
M0	22	38		
Grade			5.193798	**0.022668**
high	67	62		
low	0	5		
Whole cohort				
Age			0.317257	0.573261
>60	152	147		
≤60	51	56		
Gender			2.50239	0.113674
male	143	157		
female	60	46		
Subtype			15.606417	**0.000078**
Papillary	46	84		
Non-Papillary	153	118		
pT			7.172964	**0.007401**
T3-4	142	109		
T0-2	51	71		
pN			5.465341	**0.019397**
N1-3	75	53		
N0	108	128		
pM			0.579021	0.537858
M1	6	5		
M0	84	112		
Grade			11.224962	**0.000807**
high	198	184		
low	3	18		

**Table 3:** Correlations between risk score of the 12-marker-based classifier with RFS and clinicopathological characteristics in training cohort, validation cohort and whole cohort.

**Parameters**	**High risk**	**Low risk**	**Pearson *x*2**	**P**
Training cohort				
Age			0.421	0.516
>60	81	86		
≤60	31	27		
Gender			1.052	0.305
male	86	93		
female	26	20		
Subtype			0.880	0.348
Papillary	34	42		
Non-Papillary	75	71		
pT			3.823	0.0506
T3-4	72	64		
T0-2	27	43		
pN			2.379	0.123
N1-3	36	25		
N0	69	77		
pM			0.4292	0.685
M1	4	2		
M0	62	55		
Grade			0.000255	0.987
high	105	106		
low	6	6		
Validation cohort				
Age			0.175	0.676
>60	39	41		
≤60	17	15		
Gender			0.676	0.411
male	37	41		
female	19	15		
Subtype			0.00433	0.948
Papillary	18	18		
Non-Papillary	38	37		
pT			7.104	**0.00769**
T3-4	37	24		
T0-2	13	26		
pN			0.0504	0.822
N1-3	14	15		
N0	32	31		
pM			0.390	0.611
M1	2	1		
M0	26	28		
Grade			0.578	0.489
high	53	50		
low	3	5		
Whole cohort				
Age			0.595	0.440
>60	120	127		
≤60	48	42		
Gender			0.638	0.425
male	125	132		
female	43	37		
Subtype			0.658	0.417
Papillary	52	60		
Non-Papillary	113	108		
pT			8.317	**0.00393**
T3-4	108	89		
T0-2	41	68		
pN			0.801	0.371
N1-3	49	41		
N0	102	107		
pM			0.0421	0.837
M1	5	4		
M0	89	82		
Grade			0.213	0.645
high	158	156		
low	9	11		

### Prognostic value of lncRNAs classifiers for assessing clinical outcome

**Figure 5:**
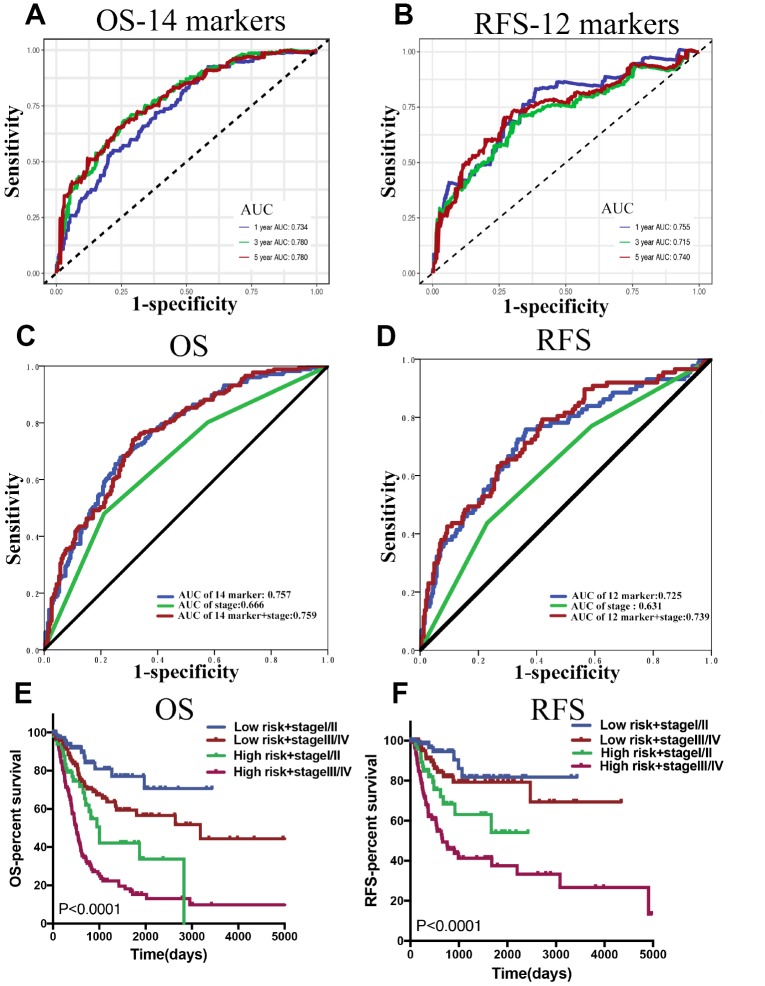
(**A** and **B**) Time dependent ROC curves at 1, 3 and 5 years, separately for OS and RFS. (**C** and **D**) The ROC for the lncRNA-score, stage, and lncRNA-score combined with stage for OS and RFS in whole BLCA cohorts. (**E** and **F**) Survival curves of BLCA patients with combinations of lncRNA-score risk and stage in the whole cohorts for OS and RFS.

In the time-dependent ROC curve analysis, the AUCs for OS (Figure 5A) in the first, third, and fifth year were 0.734, 0.78, and 0.78 respectively, while the prediction capability of the 14-lncRNA classifier was superior to the previously published lncRNA classifier [17]. As for RFS (Figure 5B), the AUCs in the first, third, and fifth year were 0.755, 0.715, and 0.740 respectively, whilst the 12-lncRNA-based classifier was mainly built to be a powerful prognostic predictor of BLCA recurrence. As shown in Table 4, the 14-marker-based classifier, age, pT, pN and pM were significantly associated with OS in the univariate Cox regression analyses. After the multivariate Cox regression analyses of the above-mentioned factors, only the 14-marker-based classifier model was retained to be a dependable and independent prognostic factor for OS (*p* < 0.001) in whole cohort. In univariate Cox regression analyses, the 12-marker- based classifier, subtype, pT, pN and pM were significantly associated with RFS in Table 5. Finally, the multivariate Cox regression analyses revealed that only the 12-marker-based classifier model could be a novel and independent prognostic factor for RFS (*p*= 0.001) in whole cohort.

**Table 4:** Univariate and multivariate Cox regression analysis of the 14-marker-based classifier with OS in training cohort, validation cohort and whole cohort.

**Parameters**	**Univariate COX**		**Multivariate COX**	
	**HR (95% CI)**	**P**	**HR (95% CI)**	**P**
Training cohort				
Age (>60 vs ≤60)	1.506(0.937,2.421)	0.090459	0.910(0.399,2.076)	0.823185
Gender(male vs female)	0.934(0.620,1.406)	0.742189		
Subtype (Papillary vs Non-Papillary)	0.780(0.512,1.189)	0.248073	1.043(0.508,2.142)	0.909174
pT (T3-4 vs T0-2)	1.654(1.066,2.564)	**0.024634**	1.269(0.513,3.138)	0.605957
pN (N1-3 vs N0)	2.153(1.451,3.196)	**1.41E-04**	1.599(0.834,3.066)	0.157505
pM (M1 vs M0)	1.969(0.270,14.378)	0.504059		
Grade(high vs low)	1.998(0.491,8.129)	0.333785		
14-marker-based classifier (high risk vs low risk)	3.994(2.629,6.068)	**8.66E-11**	5.215(2.502,10.869)	**0.00001**
Validation cohort				
Age (>60 vs ≤60)	3.135(1.595,6.165)	**0.000923**	2.766(1.286,5.948)	**0.009202**
Gender(male vs female)	0.755(0.442,1.291)	0.30446		
Subtype (Papillary vs Non-Papillary)	0.463(0.236,0.911)	**0.025825**	0.706(0.325,1.533)	0.378535
pT (T3-4 vs T0-2)	4.020(1.904,8.487)	**0.000264**	3.014(1.222,7.433)	**0.016621**
pN (N1-3 vs N0)	2.338(1.352,4.042)	**2.37E-03**	1.218(0.664,2.236)	0.523547
pM (M1 vs M0)	4.864(1.961,12.066)	0.000642		
Grade(high vs low)	21.188(0.019,23176.048)	0.39241		
14-marker-based classifier (high risk vs low risk)	2.588(1.526,4.387)	**4.16E-04**	2.005(1.091,3.685)	**0.025003**
Whole cohort				
Age (>60 vs ≤60)	1.897(1.287,2.794)	**0.001206**	1.604(0.799,3.223)	0.184
Gender(male vs female)	0.88(0.635,1.217)	0.439		
Subtype (Papillary vs Non-Papillary)	0.655(0.459,0.933)	**0.018962**	0.992(0.541,1.82)	0.98
pT (T3-4 vs T0-2)	2.14(1.472,3.111)	**0.000067**	1.489(0.745,2.978)	0.26
pN (N1-3 vs N0)	2.268(1.656,3.105)	**3.29E-07**	1.248(0.718,2.17)	0.432
pM (M1 vs M0)	3.305(1.579,6.915)	**0.001507**	1.612(0.589,4.413)	0.352
Grade(high vs low)	2.926(0.724,11.829)	0.131854		
14-marker-based classifier (high risk vs low risk)	3.526(2.537,4.901)	**6.26E-14**	3.976(2.192,7.211)	**6.00E-06**

**Table 5:** Univariate and multivariate Cox regression analysis of the 12-marker-based classifier with RFS in training cohort, validation cohort and whole cohort.

**Parameters**	**Univariate COX**		**Multivariate COX**	
	**HR (95% CI)**	**P**	**HR (95% CI)**	**P**
Training cohort				
Age (>60 vs ≤60)	2.055(1.005,4.202)	**0.048360973**	1.239(0.451,3.404)	0.678047
Gender(male vs female)	0.880(0.454,1.707)	0.704943796		
Subtype (Papillary vs Non-Papillary)	1.357(0.733,2.510)	0.331186056		
pT (T3-4 vs T0-2)	2.337(1.166,4.685)	**0.016743034**	1.636(0.635,4.212)	0.307782
pN (N1-3 vs N0)	2.576(1.482,4.477)	**0.00079688**	1.467(0.624,3.449)	0.379187
M (M1 vs M0)	6.003(1.757,20.512)	**0.004255841**	3.330(0.384,28.905)	0.275237
Grade(high vs low)	2.135(0.294,15.528)	0.453562546		
12-marker-based classifier (high risk vs low risk)	5.607(2.885,10.898)	**0.000000368**	3.364(1.349,8.384)	**0.00924**
Validation cohort				
Age (>60 vs ≤60)	0.581(0.286,1.180)	0.133271407		
Gender(male vs female)	1.124(0.527,2.399)	0.761624713		
Subtype (Papillary vs Non-Papillary)	0.341(0.130,0.891)	**0.028126777**	0.492(0.099,2.437)	0.384909
pT (T3-4 vs T0-2)	2.379(1.003,5.646)	**0.049252702**	34614.538(0,4.777E+157)	0.953672
pN (N1-3 vs N0)	2.792(1.227,6.352)	**0.014351444**	1.644(0.433,6.247)	0.466
M (M1 vs M0)	6.121(0.684,54.771)	0.105160081	4.189(0.334,52.541)	0.26697
Grade(high vs low)	22.506(0.029,17274.179)	0.35827		
12-marker-based classifier (high risk vs low risk)	2.941(1.353,6.394)	**0.006477803**	9.857(1.212,80.2)	**0.032403**
Whole cohort				
Age (>60 vs ≤60)	1.168(0.724,1.883)	0.525022		
Gender(male vs female)	0.986(0.603,1.614)	0.956337		
Subtype (Papillary vs Non-Papillary)	0.58(0.346,0.969)	**0.038**	0.694(0.322,1.494)	0.351
pT (T3-4 vs T0-2)	2.319(1.351,3.981)	**0.00229**	1.835(0.661,5.095)	0.244
pN (N1-3 vs N0)	2.647(1.681,4.17)	**0.000027**	1.537(0.769,3.072)	0.224
M (M1 vs M0)	5.815(2.003,16.885)	**0.001208**	3.808(0.809,17.927)	0.091
Grade(high vs low)	4.044(0.561,29.136)	0.165449		
12-marker-based classifier (high risk vs low risk)	4.212(2.552,6.953)	**1.88E-08**	3.816(1.698,8.571)	**0.001**

In clinical practice, the most commonly used risk classification is TNM staging. Therefore, the association between the lncRNA-based classifier models and TNM staging was explored. The ROC curve analysis compared TNM staging with the lncRNA-based classifier models which had an obvious better predictive accuracy. The results indicated that the combination of the lncRNA-based classifier models and TNM staging could enhance the ability to predict prognosis of survival and recurrence (Figure 5C, 5D). The Kaplan–Meier curves revealed that patients separated by combining the lncRNA-based risk scores and TNM staging had evidently discrepant prognoses (*p*< 0.0001, Figure 5E, 5F).

## DISCUSSION

Patients with BLCA, especially muscle-invasive bladder cancer (MIBC), still have significant risks of relapse and death, in spite of radical cystectomy [4, 6, 18, 19]. To a certain extent, the aggressiveness of BLCA cannot be accurately stratified by the TNM staging system, which mostly depends on the pathological staging without any molecular biological features [20, 21]. On that account, finding new and effective prognostic biomarkers is critical for patients with MIBC due to the disappointing clinical outcomes.

Increasing evidence has demonstrated that dysregulated lncRNAs may contribute to cancer initiation, progression and metastasis [22]. Several lncRNA-based signatures have been applied to predict the risk of cancer progression in patients with different cancer types, such as renal cell carcinoma [14] and colon cancer [15]. As for BLCA, although the prognostic value of lncRNAs has also been explored by some authors [17, 23], there are still many things to be improved. The reasons for this are the following: (1) the internal validation dataset is needed to validate the stability of the constructed model; (2) the comparison between the constructed model and the existing TNM staging system is indispensable; (3) the prognostic value of BLCA recurrence should be further explored. Therefore, in this study, based on a TCGA-BLCA cohort, we established and validated novel prognostic lncRNA-based signatures for OS and RFS, in order to improve the prediction of mortality and disease recurrence. The LASSO-Cox regression mode, as a popular tool for regression with high-dimensional predictors, has previously been performed in the study of colon cancer but has not been applied yet to the study of BLCA. Thus, in this study, the LASSO-Cox regression mode was applied as an effort to optimally select lncRNAs with high expression variances, significant prognostic values and low correlation by using LASSO penalization. A 14-lncRNA-based classifier for OS and a 12-lncRNA-based classifier for RFS were constructed and validated to optimize the predictive ability of prognosis for BLCA patients. The results indicated that the two classifiers could successfully divide BLCA patients into high/low-risk groups with significant differences in OS and RFS in training cohorts. The prognostic value of the two classifiers could be confirmed in validation cohorts, indicating the repeatability and practicability of the two lncRNA-based classifiers for the prognostic prediction for OS and RFS. As shown in Table 2 and Table 3, the 14-marker-based classifier, age, pT, pN and pM were significantly associated with OS, while the 12-marker-based classifier, subtype, pT, pN and pM were significantly associated with RFS in univariate Cox regression analyses. In multivariate Cox regression analyses, only the 14-lncRNA-based classifier model was retained to be a dependable and independent prognostic factor for OS (*p* < 0.001) and only the 12-lncRNA-based classifier model could qualify as a novel and independent prognostic factor for RFS (*p* = 0.001). In clinical practice, the most used risk classification is TNM staging. Next, the association between the lncRNA-based classifier models and TNM staging were explored. In the ROC curve analysis, compared TNM staging, the lncRNA-based classifier models had an obviously better predictive accuracy, and the combination of the lncRNA-based classifier models and TNM staging could enhance the ability to predict prognosis of survival and recurrence. The Kaplan–Meier curves revealed that patients separated by both the lncRNA-based risk scores and TNM staging had evidently discrepant prognoses.

Our study has showed that the 14-lncRNA-based classifier for OS and the 12-lncRNA-based classifier for RFS were both strongly associated with the prognosis of BLCA. However, most of the lncRNAs in our classifiers have not been completely clarified and functionally annotated. On the other hand, several lncRNAs used in our classifiers have been explored in previous studies. MAFG-AS1 has been shown to function as a ceRNA to increase the expression of MMP15 and NDUFA4. It does so by competing for miR-339-5p and miR-147b, thus exerting its oncogenic function in non-small- cell carcinoma [24] and colorectal cancer [25]. LINC01138 induces malignancies via activating arginine methyltransferase 5 and interacting with PRMT5 to promote SREBP1-mediated lipid desaturation individually in hepatocellular carcinoma [26] and clear cell renal cell carcinoma [27]. Given their strong relevance to prognosis, these genes should be explored in the future, especially in relation to BLCA.

Inevitably, the present study has some innate limitations which need to be addressed. Firstly, the current study was of a retrospective nature, since it was based on data from TCGA dataset without validating it in a prospective clinical trial. Secondly, the mechanism behind the lncRNAs in our classifiers remains entirely unclear. Hence, the need for further studies of the specific lncRNAs is indisputable, as they can contribute to a distinct understanding of the implication of lncRNAs in BLCA initiation and progression. Moreover, the information regarding several important clinicopathological features, such as treatments, was not available in the TCGA-BLCA cohort. Despite these drawbacks, the results demonstrate that our lncRNA-based classifiers could be used as reliable prognostic predictors of BLCA survival and recurrence.

In summary, a 14-lncRNA-based classifier for OS and a 12-lncRNA-based classifier for RFS were constructed using the LASSO Cox regression model. These classifiers could be novel and independent prognostic factors for OS and RFS respectively, while optimizing the predictive ability of the current (TNM) staging system. Nevertheless, future, large-scale, multi-center studies are necessary to confirm our results before the lncRNA-based signatures can be applied in the clinic.

## MATERIALS AND METHODS

### Patient datasets

TCGA-BLCA RNA sequencing dataset and corresponding clinical characteristics of patients were downloaded from the TCGA website (https://cancergenome.nih.gov/), including 414 BLCA tissues and 19 adjacent non-tumor tissues. The RFS data was downloaded from the UCSC Xena website (https://xena.ucsc.edu/). We excluded the lncRNA whose expression (read counts) was “zero” in 90% of the BLCA patients.

### Data processing

BLCA data were annotated by Gencode (GENCODE v 26) GTF file in this study. As shown in Figure 1, we used edgeR for the entire data in order to identify the differentially expressed lncRNAs(DElncRNAs) with |logFC| >1 and padj < 0.05 between tumor and normal samples. Meanwhile, we conducted a univariate Cox regression for all lncRNAs in cancer samples and chose the lncRNAs with *p* < 0.05 for the next analysis. The DElncRNAs with |logFC| >1 and padj < 0.05 were retained to determine their overlap with lncRNAs with *p* < 0.05 in the univariate Cox regression. Afterwards, these samples were randomly split into training and validation sets at a 2:1 ratio. Following this, we applied the LASSO Cox selection method at 20-fold cross-validation to construct the survival-predicting models. The predictive ability of the model for the training, validation and whole cohorts were evaluated by the Kaplan–Meier log-rank test, Time-dependent ROC curve analysis and multivariate Cox regression analysis.

### Construction of lncRNAs signature and statistical analysis

The lncRNAs-based prognosis risk score was constructed based on a linear combination of the expression level multiplied regression model (β) and the LASSO Cox selection method [28–30] at 20-fold cross-validation. Based on the cut-off of the median risk score, BLCA patients were divided into high- and low-risk groups. The Kaplan-Meier survival curves for the cases predicted to have low or high risk were produced. All the analyses were implemented in SPSS version 23.0 or R version 3.5.2 with the following packages: ‘edgeR’, ‘glmnet’, ‘survivalROC’ and ‘gplot’. All the hypotheses were two-sided and P < 0.05 was considered statistically significant.
